# Different Repellents for *Aedes aegypti* against Blood-Feeding and Oviposition

**DOI:** 10.1371/journal.pone.0103765

**Published:** 2014-07-31

**Authors:** Ali Afify, Bérénice Horlacher, Johannes Roller, C. Giovanni Galizia

**Affiliations:** 1 Neurobiology, University of Konstanz, Konstanz, Germany; 2 Heinrich Suso Gymnasium, Konstanz, Germany; New Mexico State University, United States of America

## Abstract

Methyl N,N-dimethyl anthranilate (MDA), ethyl anthranilate (EA) and butyl anthranilate (BA) were previously shown to repel *Aedes aegypti* mosquitoes from landing on human skin. However, the effect of these compounds on the orientation of flying mosquitoes in a choice situation and their effect on mosquito oviposition are not yet known. Here, we used a modified Y-tube olfactometer to test the effect of these compounds on the orientation of *Aedes aegypti* flying towards skin odor (human fingers), and we tested their effect on *Aedes aegypti* oviposition choice in a cage assay. In both behavioral situations we compared the effect to the well-documented repellent N,N-diethyl-meta-toluamide (DEET). MDA, EA, and DEET inhibited *Aedes aegypti* from flying towards skin odor while BA had no such effect. Conversely, MDA had no effect on oviposition while EA, BA, and DEET deterred oviposition, with the strongest effect observed for BA. Thus, we confirm that EA and DEET are generally repellent, while MDA is repellent only in a host-seeking context, and BA is deterrent only in an oviposition context. These compounds appear of potential use in mosquito control programs.

## Introduction

The role of olfactory cues in shaping mosquito behavior in response to host odor has been studied extensively [1]–[4]. In those studies, the use of olfactometers was established to monitor the orientation (attraction/repellency) of flying mosquitoes towards an odor source. In other studies, a mosquito landing approach was used in which the number of mosquitoes landing on a treated substrate (e.g. human skin) was used as indicator for attraction/repellency of the compound [5]–[7].

Here, we follow a terminology that was proposed to describe mosquito olfactory cues [8]; an “attractant” is a substance that encourages mosquitoes to make oriented flights towards the source while a “stimulant” is a substance that elicits a specific behavior (blood feeding or oviposition). Also, a “repellent” is a substance that encourages an oriented flight away from the source while a “deterrent” is a substance that inhibits a specific behavior (blood feeding or oviposition).

Methyl N,N-dimethyl anthranilate (MDA), ethyl anthranilate (EA) and butyl anthranilate (BA) are three non-toxic compounds, that were recently shown to elicit an avoidance behavior with host seeking *Ae. aegypti* mosquitoes in a caged landing assay [6]. In that assay, a human hand was inserted in a mosquito cage protected by a net while an intermediate net was treated with the substance to be tested. Thus, mosquitoes had a choice between landing and not landing on the net surface [6]. In nature, mosquitoes do not encounter only one odor choice in still air, but rather fly through turbulent air streams that might contain different choices of odors. Therefore, we tested the orientation response of flying mosquitoes when given a choice of skin odor plumes against skin odor plumes turbulently mixed with putative repellents.

Odors influencing host seeking mosquitoes may also affect oviposition. Three known mosquito repellents (diethyl phenyl acetamide, diethyl benzamide, and N,N-diethyl-meta-toluamide (DEET)) have been shown to deter three mosquito species (*Ae. aegypti*, *Ae. albopictus*, and *Culex quinquefasciatus*) from oviposition [9]. Since all three substances also act as deterrent in a host-seeking context [10], [11], this deterrent effect indicates a general repellency of these compounds towards mosquitoes throughout the gonotrophic cycle rather than a specialized, ecologically restricted effect on gravid females. Consequently, we hypothesized that MDA, EA and BA could also have a deterrent effect on the oviposition of *Ae. aegypti* mosquitoes.

In this study, we tested whether the new repellents (MDA, EA and BA), as compared to DEET, would affect (attract/repel) *Ae. aegypti* host seeking behavior in a turbulent upwind odor-choice condition using a modified Y-tube olfactometer. We also tested in oviposition cages similar to those used in the landing assay of the previous study [6], whether these compounds have a deterrent effect against *Ae. aegypti* oviposition at three different concentrations. We found that MDA, EA, and DEET repel mosquitoes from flying towards skin odor while BA had no effect. On the other hand, MDA had no effect on oviposition while EA, BA, and DEET deterred oviposition to different degrees. We suggest a weak effect for BA on host seeking mosquitoes and another use for EA, BA, and DEET in mosquito control programs as oviposition deterrents.

## Materials and Methods

### Mosquito colony

An *Ae. aegypti* colony was initiated in 2010 from eggs obtained from Biogents AG (Regensburg, Germany). Mosquitoes were raised in a climate chamber maintained at 25–28°C, 60–70% RH and L12:D12 photoperiod. After hatching, mosquito larvae were fed on fish food (TetraMin, Tetra GmbH, Melle, Germany) in water every other day. Cotton pads soaked with sugar solution (10%, w/vol) were provided to feed adult mosquitoes as a source of carbohydrates. Mosquito females were blood fed on pigeons for egg laying. The use of pigeons in blood feeding was done at the animal research facility of the university of Konstanz and approved by the authorities (Regierungspräsidium Freiburg) according to German law (TierSchG §10a, approval 35-9185.82/I). Mosquitoes were allowed to feed, through the cage net, on only the abdomen of a pigeon that was treated afterwards with anti-allergic gel (Fenestil gel, Novartis GmbH, Nürnberg, Germany) to reduce irritation.

### Orientation experiments

**Figure 1:**
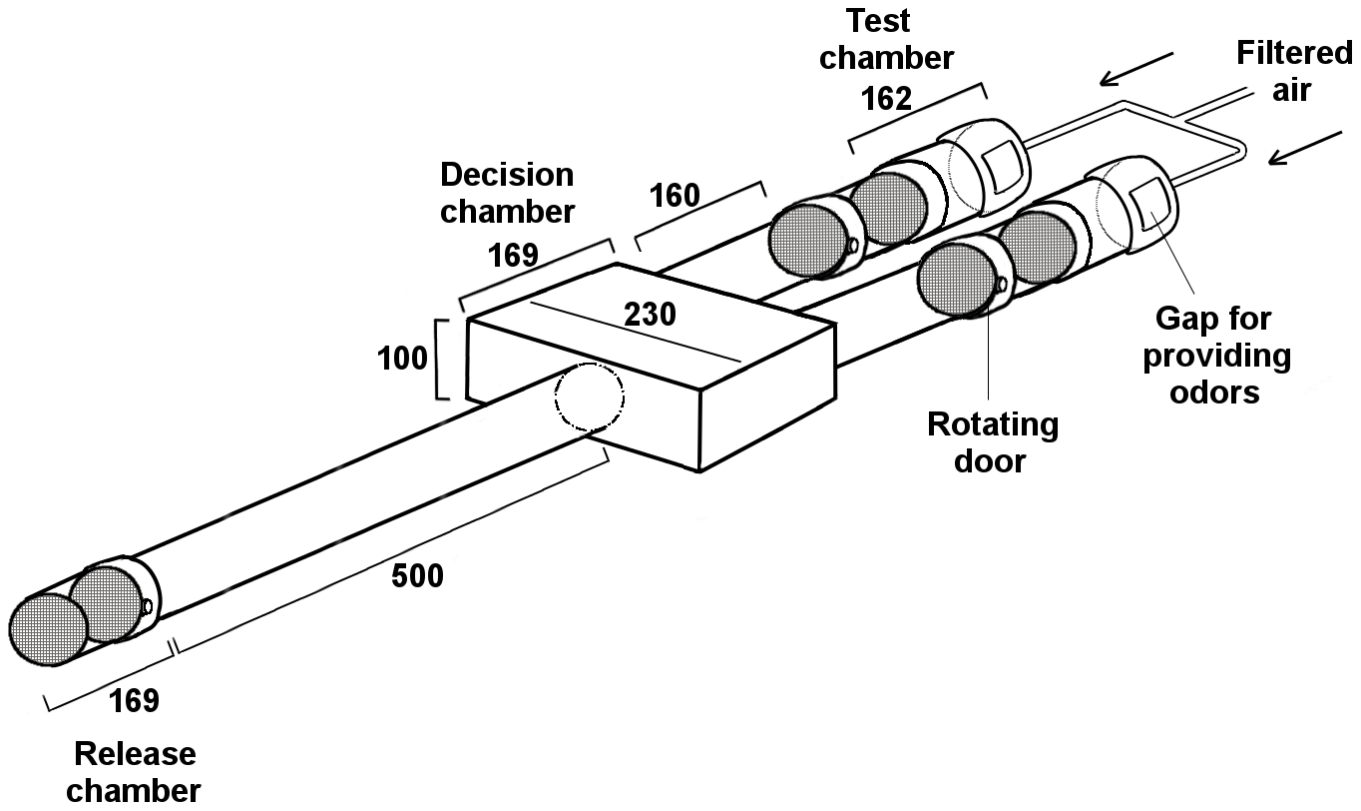
Schematic drawing of the modified Y-tube olfactometer. All dimensions are in millimeter.

A modified Y-tube olfactometer [1] was used to test the orientation response (attraction/repellence) of host seeking mosquitoes towards the test compounds. The olfactometer (Fig. 1) was made of Plexiglas and consisted of a long “stem” tube (500 mm) with a release chamber (169 mm length) attached to one end and a decision chamber to the other end. The decision chamber was a box (230×169×100 mm) that branches into two short tubes (160 mm), each ending in a trapping chamber (169 mm) and a test/control chamber (162 mm) that delivered the test odorants.

Compressed air was charcoal filtered (Ultrafilter AK 03/05. Hilden, Germany) and humidified in deionized water before it was introduced into the two test branches of the olfactometer. The incoming air temperature was kept at 23±1°C, relative humidity at 60±5% and air speed at 0.6±0.05 m/s, measured at the center of the stem tube. This wind speed was used because it encourages more mosquitoes to fly upwind.

The odors used were: Methyl N,N-dimethyl anthranilate (MDA), Aldrich, St. Louis, USA; Ethyl anthranilate (EA), Aldrich, St. Louis, USA, ≥99% purity; Butyl anthranilate (BA), SAFC, St. Louis, USA, ≥99% purity; and N,N-diethyl-meta-toluamide (DEET), Aldrich, St. Louis, USA, 97% purity. All odors were diluted to 10% in acetone (Merck, Darmstadt, Germany, ≥99.9% purity). In the case of DEET, this dilution is in the range of commercial insect repellents [12], [13]. A filter paper (VWR, Leuven, Belgium. 90 mm diameter) was folded, set in a glass dish (18 mm diameter and 24 mm depth) and soaked with 500 µl of the diluted test substance. 500 µl acetone were used as solvent control. After applying the test/control solutions to the filter papers, they were left for 5 min in a laminar flow hood to allow the acetone to evaporate before they were introduced into the test/control chamber. The experimenter provided two fingers through an opening in each chamber behind the glass dish to lure the mosquitoes upwind. The use of cosmetics was avoided at the day of the experiment and the two hands were rubbed together for 1 min before each trial to achieve homogeneity. 20 non-blood fed female *Ae. aegypti* mosquitoes, 5–10 days old, were placed in the release chamber and left for 30 seconds for acclimatization before opening a rotating door. With the door open, mosquitoes could fly upwind in response to the skin odor and enter the decision chamber. At this point they had the choice to fly towards the test or the control chamber. After 2 min, the number of mosquitoes trapped in each of the two chambers was counted.

Before testing the compounds, we tested in a preliminary experiment whether mosquitoes would choose either side (test chambers) of the olfactometer when given the same odor choice in both chambers (two fingers of an experimenter). Mosquitoes did not discriminate between the two sides (p = 0.792, Student *t*-test, n = 6) showing no side bias. We also tested whether mosquitoes could discriminate the side that contains an attractive odor (two fingers) when there is no odor in the other side (two fingers covered with a glove). Mosquitoes preferred the chamber with the attractant more than the chamber with no odor (p = 0.027, Student t-test, n = 3). This shows that mosquitoes could follow the odor plume of an attractant in the olfactometer.

Afterwards, all compounds were tested twice with two different skin odors (two experimenters). With skin odor 1 (experiment 1), each compound was tested using 5 groups of mosquitoes and each group was tested twice to confirm there was no side bias (once with the test compound in the right chamber and 5 min later with the test compound in the left chamber). With skin odor 2 (experiment 2), each compound was tested with 7 groups of mosquitoes and each group was tested only once while alternating the test compound between right and left chambers. In both experiments, four olfactometers were alternated between trials to reduce contamination.

### Oviposition experiment

Oviposition response of *Ae. aegypti* towards MDA, EA, BA, and DEET was tested using a standard cage assay [14], [15]. Unlike the orientation experiment which shows the attractant/repellent effect of an odor, this test only shows the deterrent/stimulant effect. We used 5 white plastic mosquito cages (30×30×30 cm) with three mesh sides. We placed two oviposition cups and 20 gravid females (1–2 weeks old, four days post blood feeding) into each cage. In all experiments, test cups were positioned pseudorandomly to one corner and control cups were placed diagonally in the opposite corner of the cage. To test for position bias, mosquitoes were offered four cups of clean water, one cup at each corner of the cage. Mosquitoes distributed the eggs equally in the four cups (ANOVA, p = 0.800, n = 5) showing no position bias between the different corners of the cage.

Oviposition cups were glass crystallizing dishes (VWR, Darmstadt, Germany. 50 mm diameter and 40 ml volume) filled with 30 ml of either the test or the control solution. We prepared stock solutions of the four compounds in acetone and added 1 ml of each solution to 30 ml of water to reach the indicated final concentration (1, 10, or 100 ppm) in test cups while 1 ml of acetone was added similarly to water in control cups. After adding the compounds or the pure acetone, we allowed the acetone to evaporate for 30 min before placing the cups in the cages. The longer evaporation time in this experiment as compared to the filter paper situation above was chosen because acetone in water evaporates more slowly.

Experiments started at 3–4 pm and stopped at 10 am the next morning. The total number of eggs in each cup was manually counted and the cups were then rinsed with tap water followed by acetone and reused in subsequent experiments.

### Statistical analysis

For orientation experiments, the Preference Index (PI) was used to indicate the response of mosquitoes to repellents in the olfactometer. The Preference Index is calculated as follows:
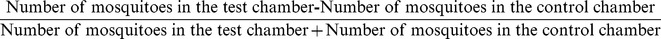
This gives values from −1 to +1, with 0 indicating neutral response, negative values indicating repellency while positive values indicate attraction.

The Preference Index was calculated for each replicate and arcsine transformed for analysis. We first tested for side bias between the first trials (when the test compound was in the right side) and the second trials (when the test compound was in the left side) using a paired *t*-test. We did not find a side bias, thus in the second experiment we did not test each group of mosquitoes twice any more.

Afterwards, we pooled all trials (arcsine transformed Preference Index) and tested the hypothesis that the mean PI for each compound is different from a 50∶50 choice (PI = 0) using Student *t*-test.

For the oviposition experiments, egg numbers were high and resulted normally distributed. We used a paired *t*-test to compare the number of eggs (actual numbers) between test and control cups.

For visualization, oviposition data were plotted as Oviposition Activity Index (OAI) as described by Kramer and Mulla [16]:
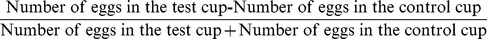
This also gives values from −1 to +1, with 0 indicating neutral response, negative values deterrence and positive values a stimulant effect.

All statistical analyses were done in R [17].

## Results

### Orientation behavior of host seeking mosquitoes

**Figure 2:**
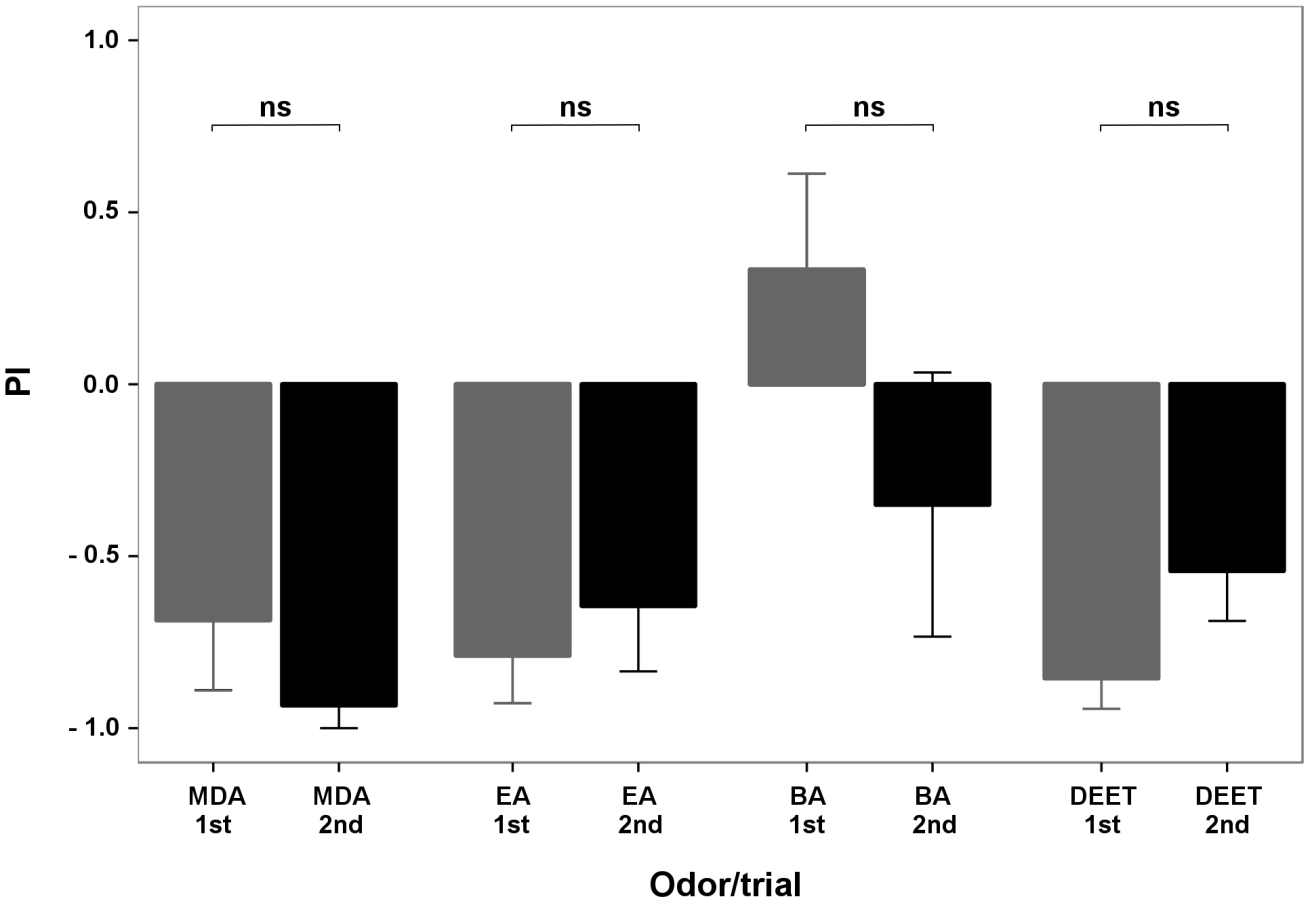
Preference Index (PI) of MDA, EA, BA, and DEET in both trials of experiment 1. The response of non-blood fed *Ae. aegypti* females to MDA, EA, BA, and DEET did not change significantly between first and second trial (p = 0.440, 0.649, 0.313, and 0.170, respectively, paired *t*-test, n = 5).

**Figure 3:**
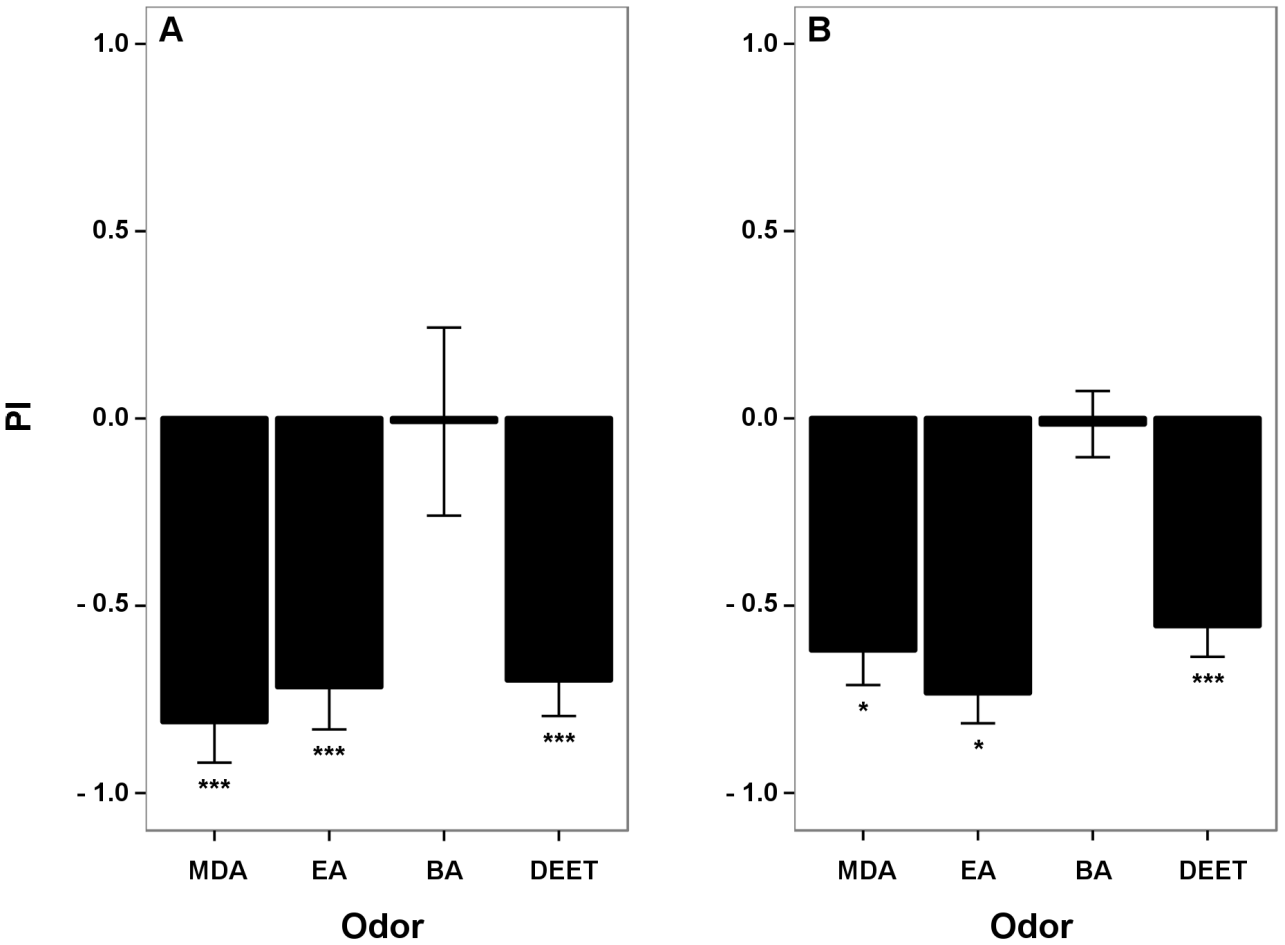
Preference Index (PI) of MDA, EA, BA, and DEET in experiment 1 and 2. Non-blood fed *Ae. aegypti* females were repelled by MDA, EA, and DEET while BA did not have an effect on mosquito orientation. **A**) Pooled preference index from first and second trial in experiment 1 shows a repellent effect for MDA, EA, and DEET (p<0.001, Student *t*-test, n = 10) and no effect for BA (p = 0.922, Student *t*-test, n = 10). **B**) Preference index in experiment 2 shows a repellent effect for MDA, EA, and DEET (p = 0.004, 0.002, and <0.001, respectively, Student *t*-test, n = 7) and no effect for BA (p = 0.868, Student *t*-test, n = 7). Number of asterisks indicates the level of significance; p<0.05 (*), and p<0.001 (***).

In experiment 1, we tested each group of mosquitoes twice to check for side bias. The proportion of mosquitoes choosing MDA, EA, BA, or DEET were not significantly different between right and left chambers (p = 0.440, 0.649, 0.313, and 0.170, respectively, paired *t*-test, n = 5 each, Fig. 2). We therefore pooled all first and second trials together (n = 10) and tested for repellency of the compounds. MDA, EA, and DEET showed significant repellency with mean PIs significantly lower than that of a neutral 50∶50 distribution between right and left chambers (p<0.001, Student *t*-test, n = 10, Fig. 3A). On the other hand, the proportion of mosquitoes that chose BA (PI) was not significantly different (p = 0.922, Student *t*-test, n = 10, Fig. 3A) from 0 (PI of a 50∶50 distribution). In this experiment, the proportion of mosquitoes that made a choice (trapped in either the test or the control chamber) when testing BA was very low (mean = 9.5%) compared with those when testing MDA, EA, and DEET (24.5, 65.5, and 39%, respectively).

Because we did not find any side bias in experiment 1, we did not continue to test each group of mosquitoes twice in experiment 2, but we nevertheless alternated the test compounds between the right and left chambers across trials. In this experiment, there was less variation in the proportion of mosquitoes that made a choice (mean = 42, 40, 39, and 43.5% when testing MDA, EA, BA and DEET, respectively).

Confirming the results of experiment 1, MDA, EA, and DEET showed significant repellency (p = 0.004, 0.002, and <0.001, respectively, Student *t*-test, n = 7 each, Fig. 3B) while BA did not repel the mosquitoes from flying into the test chamber (p = 0.868, Student *t*-test, n = 7, Fig. 3B).

### Oviposition behavior

**Figure 4:**
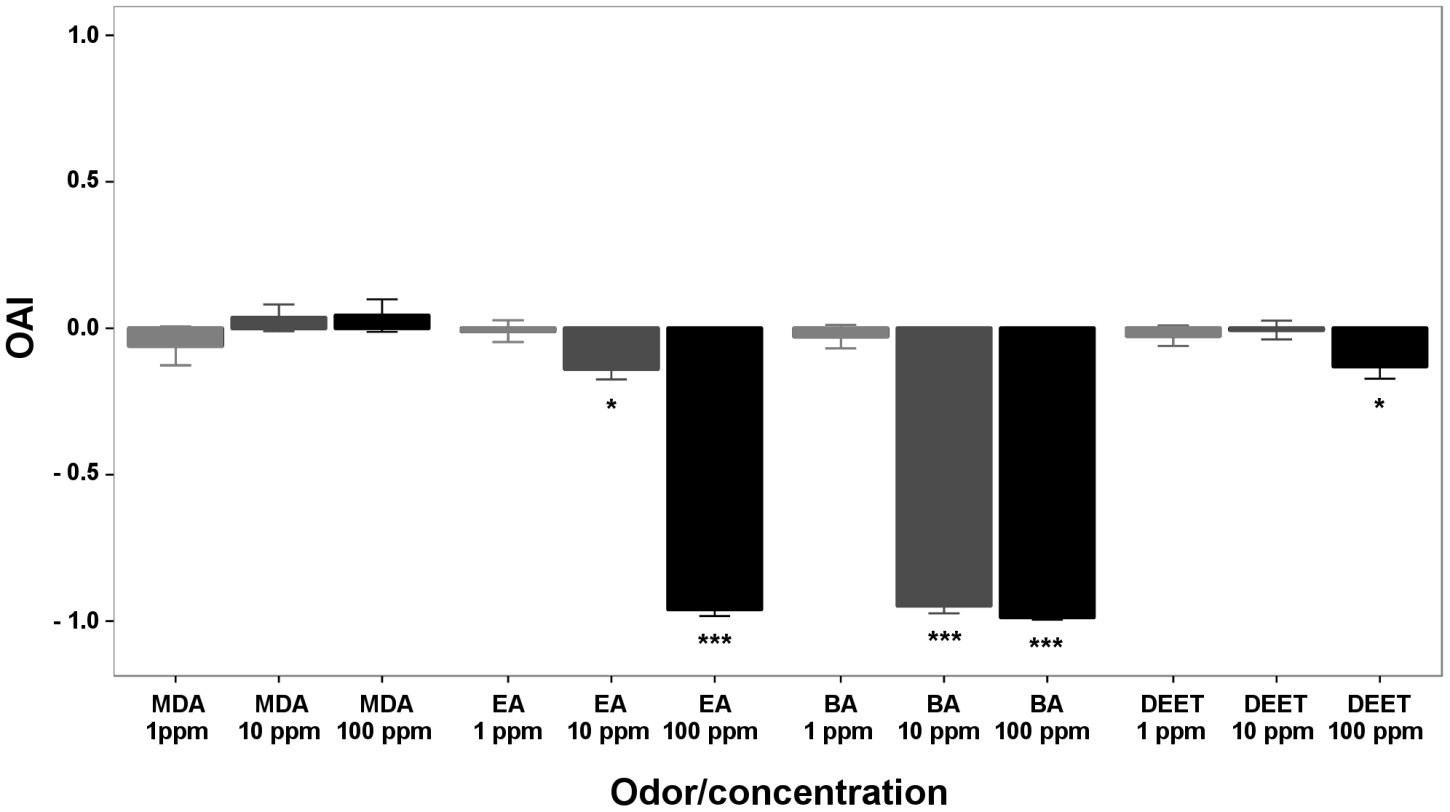
Oviposition Activity Index (OAI) of MDA, EA, BA, and DEET at three different concentrations. MDA had no effect on oviposition at 1, 10, or 100(p = 0.396, 0.472, and 0.484, respectively, paired *t*-test, n = 5). There was no effect for EA at 1 ppm but deterred oviposition at 10 and 100 ppm (p = 0.774, 0.017, and <0.001, paired *t*-test, n = 5). BA had no effect at 1 ppm but strongly deterred oviposition at 10 and 100 ppm (p = 0.513, <0.001, and <0.001, respectively, paired *t*-test, n = 5). DEET had no effect at 1 and 10 ppm and a weak deterrent effect at 100 ppm (p = 0.487, 0.837, and 0.037, respectively, paired *t*-test, n = 5). Number of asterisks indicates the level of significance; p<0.05 (*), and p<0.001 (***).

We tested the oviposition effect of the four compounds at three different concentrations (1, 10, and 100 ppm). MDA was not deterrent at any concentration (p = 0.396, 0.472, and 0.484 for 1, 10, and 100 ppm, respectively, paired *t*-test, n = 5 each, Fig. 4, Table 1). On the other hand, EA had no effect at 1 ppm but was deterrent at 10 and 100 ppm (p = 0.774, 0.017, and <0.001, respectively, paired *t*-test, n = 5 each, Fig. 4, Table 1). BA had no effect at 1 ppm (p = 0.513, paired *t*-test, n = 5, Fig. 4, Table 1) but surprisingly showed a strong oviposition deterrence at 10 and 100 ppm (p<0.001, paired *t*-test, n = 5, Fig. 4, Table 1). DEET was not deterrent at 1 or 10 ppm and only deterrent at 100 ppm (p = 0.487, 0.837, and 0.037, respectively, paired *t*-test, n = 5 each, Fig. 4, Table 1).

**Table 1:** Mean number of eggs laid on different concentrations of MDA, EA, BA, and DEET in comparison with water.

Odor	Concentration (ppm)	Mean number of eggs (SE, n)	
		Treatment	Control
MDA	1	282 (19, 5)	319 (22, 5)
	10	323(19, 5)	300 (13, 5)
	100	343 (19, 5)	315 (19, 5)
EA	1	306 (11, 5)	313 (16, 5)
	10	281 (13, 5)	372 (13, 5)
	100	11 (06, 5)	522 (16, 5)
BA	1	277 (13, 5)	294 (13, 5)
	10	13 (07, 5)	490 (08, 5)
	100	3 (02, 5)	478 (08, 5)
DEET	1	306 (11, 5)	322 (14, 5)
	10	311 (08, 5)	315 (13, 5)
	100	263 (13, 5)	342 (13, 5)

## Discussion

In a previous study, MDA, EA and BA were reported to have a repellent effect against *Ae. aegypti* mosquitoes similar in magnitude to the well-known repellent DEET [6]. In that study, an “arm-in cage” assay was used, in which mosquitoes were allowed to land close to but not touch the test compounds and the skin, suggesting an olfactory effect of the four compounds [6]. However, that assay did not provide information on whether the mosquitoes would be affected by plumes of these odors while flying through a moving air stream or not. Here, we show using a two-choice Y-tube olfactometer, that MDA, EA, and DEET influenced the orientation of flying *Ae. aegypti* mosquitoes. Host seeking mosquitoes could detect these odors in the air at the decision chamber and orient themselves away from the source. On the other hand, BA which previously showed a strong repellent effect in arm-in cage assays [6] did not influence mosquito orientation in the olfactometer. It is worth to note that when testing BA in the first experiment, the percentage of mosquitoes that made a choice was very low (9.5%) but it was comparable to the other substances in the second experiment (39%). We do not know why the batch of mosquitoes tested for BA in the first experiment was unresponsive. Odors from spatially separate sources remain perceptually separate over long distances for insects, since their plumes do not fuse uniformly [18], [19]. The fact that we found a repellent effect of these substances in a situation of turbulent air, and with separate odor sources (fingers and odor filter paper) argues that the repellent effect is a direct response to the odor, rather than a mixture effect between repellent and skin odor (e.g. a masking effect due to creation of olfactory blends).

In addition, we tested the four compounds for their oviposition effect on *Ae. aegypti* gravid females. In preliminary experiments, we could not stimulate the gravid females to fly through the Y-tube olfactometer (data not shown). Therefore, we used the end point of mosquito oviposition behavior (number of eggs) as an indicator for the effect of these compounds on oviposition in cage assay; gravid females had the opportunity to land and touch the surface of the test solutions before making a decision. Using this assay, we show an inhibitory effect on oviposition, but we do not know whether it is an olfactory or a gustatory effect. We show that MDA had no effect on the oviposition of *Ae. aegypti* at any of the tested concentrations. EA and BA also had no effect at 1 ppm but inhibited oviposition at 10 and 100 ppm. On the other hand, DEET showed a weak deterrent effect already at 100 ppm, thus resulting more potent than in a previous study that showed a deterrent effect for DEET at 1000 ppm but not below [9]. However, though statistically significant, this effect was not large, and may be irrelevant in a field situation.

MDA was repellent against host seeking mosquitoes but had no effect on gravid females, at least at the tested concentrations. One reason could be that the effect of MDA is specific for host seeking behavior. It has been shown that olfactory responsiveness of female mosquitoes towards host odor is reduced following blood feeding [20]–[22]. This reduced response was attributed to down-regulation of odorant receptor expression after a blood meal [23]. MDA could therefore be selectively repellent for host seeking behavior due to a sensitivity change through the gonotrophic cycle of the female mosquito. Another reason could be that unlike other repellents, MDA might not have a strong repellent smell *per se* but rather a smell that masks or neutralizes skin odor even in a turbulent environment. In this case, gravid females would not have a preference for egg laying on clean water over water with MDA.

EA and DEET were the only odors that interfered both with host seeking behavior and oviposition. The two compounds appear to possess a general repellent effect against *Ae. aegypti* mosquitoes. In addition, unlike odors that convey an ecologically relevant message (e.g. odors of known mosquito predators that deter oviposition [24]), these compounds are not known to have been associated with natural habitats in mosquito evolution. Therefore, the reason for mosquito avoidance of these compounds remains to be elucidated.

BA had no effect on host seeking flying mosquitoes, but showed the strongest oviposition deterrence. Given that BA was reported as a repellent in an arm-in cage host seeking paradigm [6], we conclude that BA has a general repellent effect only in still air situations when mosquitoes are allowed to land on (or above) a treated surface, whether this surface is an oviposition substrate or human skin.

In conclusion, our results together with others suggest that the compound MDA is a host seeking repellent. EA on the other hand is a general repellent comparable to DEET, while BA inhibits oviposition and acts as a limited repellent in a host-seeking context. These substances could be integrated in mosquito control programs, either against host seeking (MDA) or egg laying behavior (BA) or both (EA). Future studies, in which field or semi-field experiments are used, need to confirm the potential of these compounds against mosquitoes in nature.
